# Algorithm in the Diagnosis of Febrile Illness Using Pathogen-specific Rapid Diagnostic Tests

**DOI:** 10.1093/cid/ciz665

**Published:** 2019-07-17

**Authors:** Sunil Pokharel, Lisa J White, Ricardo Aguas, Olivier Celhay, Karell G Pellé, Sabine Dittrich

**Affiliations:** 1 Centre for Tropical Medicine and Global Health, Nuffield Department of Medicine, University of Oxford, United Kingdom; 2 Foundation for Innovative New Diagnostics, Geneva, Switzerland; 3 Mahidol-Oxford Tropical Medicine Research Unit, Faculty of Tropical Medicine, Mahidol University, Bangkok, Thailand

**Keywords:** acute undifferentiated febrile illness, diagnosis, algorithm, rapid diagnostic test

## Abstract

**Background:**

In the absence of proper guidelines and algorithms, available rapid diagnostic tests (RDTs) for common acute undifferentiated febrile illnesses are often used inappropriately.

**Methods:**

Using prevalence data of 5 common febrile illnesses from India and Cambodia, and performance characteristics (sensitivity and specificity) of relevant pathogen-specific RDTs, we used a mathematical model to predict the probability of correct identification of each disease when diagnostic testing occurs either simultaneously or sequentially in various algorithms. We developed a web-based application of the model so as to visualize and compare output diagnostic algorithms when different disease prevalence and test performance characteristics are introduced.

**Results:**

Diagnostic algorithms with appropriate sequential testing predicted correct identification of etiology in 74% and 89% of patients in India and Cambodia, respectively, compared with 46% and 49% with simultaneous testing. The optimally performing sequential diagnostic algorithms differed in India and Cambodia due to varying disease prevalence.

**Conclusions:**

Simultaneous testing is not appropriate for the diagnosis of acute undifferentiated febrile illnesses with presently available tests, which should deter the unsupervised use of multiplex diagnostic tests. The implementation of adaptive algorithms can predict better diagnosis and add value to the available RDTs. The web application of the model can serve as a tool to identify the optimal diagnostic algorithm in different epidemiological settings, while taking into account the local epidemiological variables and accuracy of available tests.

## Teaser

Exploring various approaches for the use of multiple rapid diagnostic tests in diagnosis of acute undifferentiated febrile illness using a mathematical model, we found geographically explicit sequential testing algorithms accounting the local epidemiology and performance characteristics of available tests to be more efficient in diagnosis.

The diagnosis of acute undifferentiated febrile illnesses (AUFIs)—commonly caused by a diversity of organisms such as *Plasmodium* species, dengue virus, *Salmonella enterica* serovar Typhi, *Salmonella enterica* serovar Paratyphi A, *Orientia tsutsugamushi*, *Rickettsia typhi*, *Leptospira* species, chikungunya virus, yellow fever virus, *Burkholderia pseudomallei*, *Brucella* species, and self-limiting viral infections—is an immense challenge in low-resource settings in Asia and Africa [1–4]. These organisms, although clinically “undifferentiated,” are unique in terms of their clinical course and need proper identification for appropriate treatment.

The introduction of quality malaria rapid diagnostic tests (RDTs) at the point of care (POC) has improved the diagnosis and management of malaria dramatically [5]. However, a large fraction of AUFIs, even in malaria-endemic areas, is attributable to nonmalarial infections [6]. The World Health Organization currently recommends the use of malaria RDTs in the diagnosis of fever and if negative, assessment for other causes is recommended, albeit not further specified. Often, in cases where patients present with a fever, they are treated empirically with antimalarial drugs in malaria-endemic regions and with antibiotics in nonmalaria-endemic regions, or often with both [7].

Developments in rapid test platforms in the past few decades have produced commercially available RDTs for some of the causes of nonmalarial febrile illnesses [8]; however, these tests are limited by suboptimal accuracies [9–11]. Currently, multiplex POC tests that can measure multiple antigens/antibodies for different diseases simultaneously from a single sample are being explored [12]. The current commercially available RDTs, despite their suboptimal accuracies, can contribute to improved patient care when used appropriately relying on clinical algorithms and protocols [13]. With the increasing availability and ongoing developments of singular and multiplex RDTs, protocols to guide their proper utilization are needed.

The aim of this study was to develop a mathematical model framework and apply it to 2 different epidemiological settings, India and Cambodia, countries with similar common disease etiologies for AUFIs but with different prevalence of diseases [14, 15]. We tested the diagnostic prediction of available RDTs when applied simultaneously vs sequentially in various algorithms in both settings to explore the optimal diagnostic approach with currently available RDTs. We also developed a web application of the model that can serve as a tool to identify the optimal diagnostic algorithm in different epidemiological settings, taking into account the local epidemiology and accuracy of available tests.

## MATERIALS AND METHODS

### Definitions

#### Simultaneous Testing

**Figure 1.**
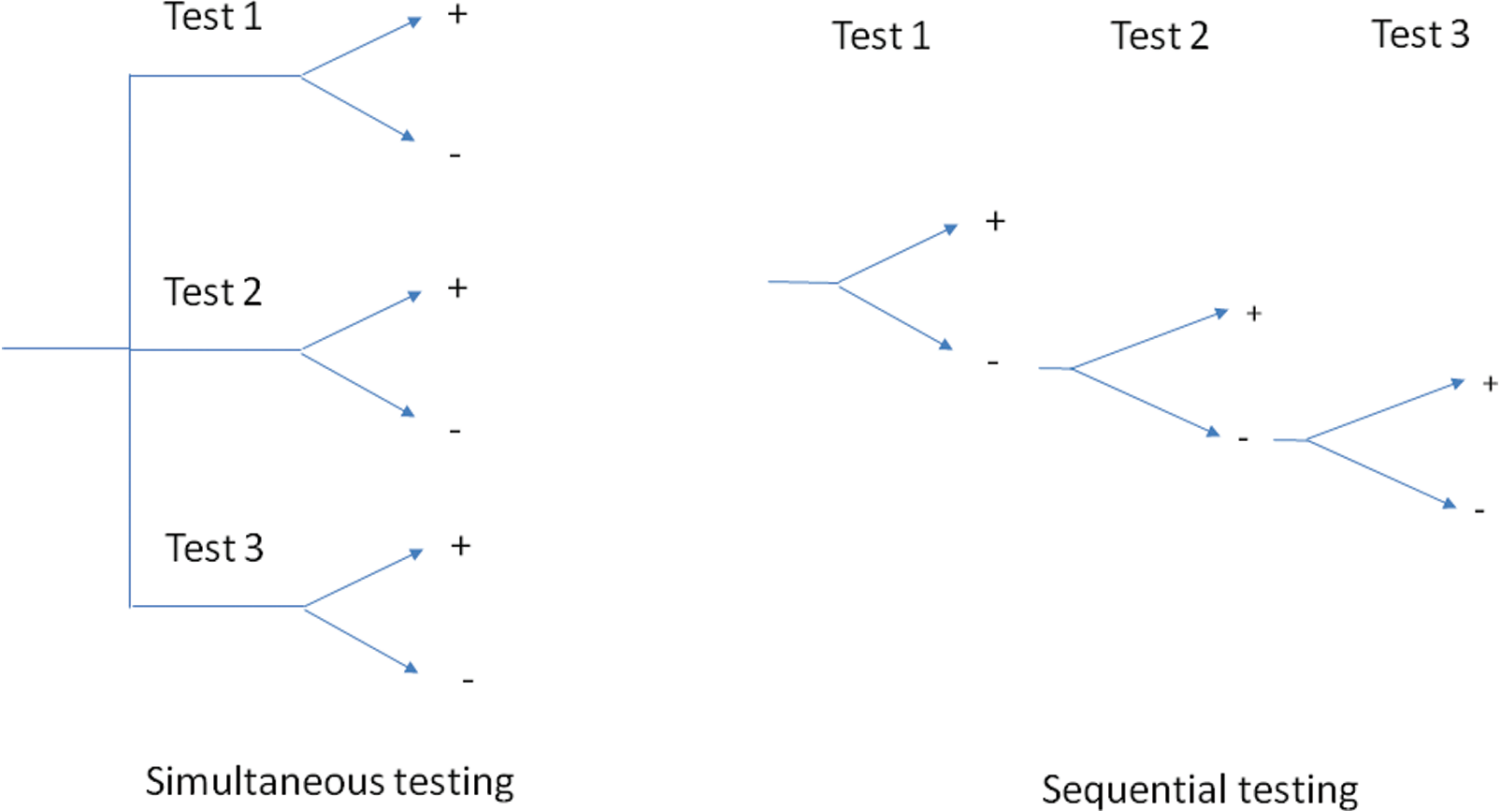
Results of simultaneous vs sequential testing.

Simultaneous testing is defined as tests being applied all at the same time (Figure 1). This is applicable to multiplexed rapid diagnostic testing or testing in off-site reference laboratories where multiple tests are subjected to a sample.

#### Sequential Testing

In sequential testing, tests are applied in a sequential manner until one provides a positive result (Figure 1). This is done with single RDTs performed at the POC. In a case where a multiplex RDT is to be used, reading the result in sequence corresponds to sequential testing.

#### Correct Diagnosis

Correct diagnosis (CD) is defined as correctly identifying the etiology for the disease and excluding the ambiguous multiple positive results. CD corresponds to the true positives in sequential testing and includes a single true-positive result, indicating a clear-cut correct identification of etiology and ignoring inconclusive multiple positive results occurring in simultaneous testing.

In routine clinical practice, it is not possible to differentiate true-positive from false-positive results by reading the test panel with multiple positive results. In the absence of further laboratory tests and clinical expertise to ascertain the etiology of a disease in a resource-poor setting, empirical treatment decisions are made leaning towards the most prevalent etiology. CD that offers a clear-cut correct identification of etiology with a single positive test result is a pertinent measure of useful diagnostic testing, especially where pathogen identification relies solely on test results.

#### Correct Diagnosis Score

The CD score is defined as the total number of correctly diagnosed patients out of the total population tested.

$$\begin{array}{l} Correct\;diagnosis\;score=\frac{Correct\;diagnosis}{Population\;tested}. \end{array}$$

#### Optimal Algorithm

Optimal algorithm is defined as the sequential testing in a particular order that gives the highest correct diagnosis score.

### Data

Prevalence data for common AUFIs were derived from the preliminary report of an ongoing febrile illness surveillance recruiting 27 586 patients in India [14] and a prospective study recruiting 1193 patients in Cambodia [15]. The RDT performance characteristic data were derived from the latest Cochrane reviews on malaria and typhoid RDTs [10, 16] and from other relevant sources for dengue, scrub typhus, and leptospirosis [9, 11, 17] (Table 1).

**Table 1.** Model Inputs: Acute Febrile Illness Disease Prevalence in India and Cambodia and Sensitivities and Specificities of Rapid Diagnostic Tests

Disease	Disease Prevalence, %		RDTs		
	India [14]	Cambodia [15]	RDT Name, Analyte	Sensitivity, % (95% CI)	Specificity, % (95% CI)
Malaria	3	31.8^a^	NA, HRP-2 by itself or with aldolase/pLDH (average estimate) [16]	95 (93.5–96.2)	95.2 (93.4–99.4)
Dengue	7	3.5	SD Bioline Dengue duo RDT, dengue virus NS1 + IgM [17]	84.2 (75.5–92.9)^b^	94.4 (88.8–100)^b^
Scrub typhus	4	2.1	Scrub typhus PanBio ICT, *Orientia tsutsugamushi* IgM [9]	72.8 (57.8–83.8)	96.8 (91.7–99.7)
Typhoid fever	1	0.1	Test-It Typhoid kit, IgM against *Salmonella* Typhi LPS 09 [10]	69 (59–78)	90 (78–93)
Leptospirosis	4	3.7	*Leptospira* Test-It, *Leptospira* IgM [11]	71 (41.9–91.6)	64.6 (59.8–69.3)

Abbreviations: CI, confidence interval; HRP-2, histidine-rich protein 2; IgM, immunoglobulin M; LPS, lipopolysaccharide; NA, not applicable; NS1, nonstructural protein 1; pLDH, plasmodium lactate dehydrogenase; RDT, rapid diagnostic test.

^a^Pathogen prevalence = 45.5%; fraction attributable to disease among positive tests = 70%; adjusted disease prevalence = 45.5 × 0.7 = 31.8.

### Model Assumptions

1. At the time of testing, each individual was infected with only 1 disease.
2. The likelihood that individuals test positive for any disease depended on test characteristics and disease status and not any other individual covariate.
3. Only patients with diseases specified in Table 1 were subjected to testing. Patients with other prevalent infections in the study setting were not tested.

### Model

A stochastic model using Monte Carlo simulation in R statistical software version 3.5.0 [18] was applied to examine the accuracy of combinations of diagnostic tests in predicting the true cause of fever given the diagnostic performance and disease prevalence for a particular setting. The model accounts for 5 diseases for which commercial RDTs with previously described sensitivity and specificity are available.

Each simulated patient was assigned a disease by comparing disease prevalence with random number drawn from uniform probability function. This provided the pretest diagnosis or the disease assigned to a patient before being subjected to any test. The true-positive, false-positive, true-negative, and false-negative diagnoses for each patient were predicted by comparing test accuracies with the random numbers from the uniform probability function. The code to run the simulation and generate outputs is provided in the Supplementary Materials.

We applied the model for the application of tests simultaneously and sequentially in all possible algorithms.

The number of possible algorithms in sequential testing is 5! = 120
Simultaneous testing = 1

### Model Output

We predicted the correct diagnosis scores for the simultaneous testing approach, and each algorithm under the sequential testing approach. We further predicted the proportion of patients correctly diagnosed for each disease as well as the predictive values for each test. The mathematical interaction between disease prevalence and diagnostic test accuracies in predicting the correct diagnosis and predictive values is provided in the Supplementary Materials.

### Web-based Application

We applied the same methods to develop a web-based application using R [18] and the web application framework R package “shiny” [19]. The web application runs 10 000 simulations of each algorithm and outputs the predicted correct diagnoses and positive predictive values (PPVs) for each of those algorithms, highlighting the test sequence for the optimally performing ones (https://moru.shinyapps.io/diagnostic-algorithm-app/).

## RESULTS

We ran 100 trials of 10 000 simulations in simultaneous testing and in each sequential testing algorithm, outputting the predicted correct diagnoses and predictive values for each.

### Simultaneous Testing Versus the Optimal Sequential Testing Algorithm

Applying all the tests simultaneously in the entire population resulted in predicted correct diagnoses of only 46% and 49% in the populations of India and Cambodia, respectively. However, the optimal algorithm when the tests were applied sequentially produced a correct diagnosis of 74% and 89%, respectively. The proportion of each disease correctly identified increased substantially using sequential testing in both settings, except for typhoid fever, which remained low. The highest increase was observed for dengue in India where the proportion of correctly diagnosed patients increased from 45% to 84%, and malaria in Cambodia where an increase was observed from 50% to 95% (Table 2).

**Table 2.** Output Metrics Comparing Simultaneous Testing and Optimal Sequential Testing Algorithm

Output Metrics	Simultaneous Testing		Optimal Sequential Testing	
	India	Cambodia	India	Cambodia
Correct diagnosis scores	0.46	0.49	0.74	0.89
Proportion of malaria cases correctly diagnosed	0.50	0.50	0.86	0.95
Proportion of dengue cases correctly diagnosed	0.45	0.45	0.84	0.78
Proportion of scrub typhus cases correctly diagnosed	0.38	0.38	0.69	0.69
Proportion of typhoid cases correctly diagnosed	0.38	0.38	0.38	0.38
Proportion of leptospirosis cases correctly diagnosed	0.55	0.55	0.61	0.61
PPV malaria test	0.79	0.99	0.89	0.99
PPV dengue test	0.89	0.57	0.89	0.89
PPV scrub typhus test	0.86	0.55	0.91	0.84
PPV typhoid test	0.28	0.02	0.62	0.13
PPV leptospirosis test	0.35	0.17	0.70	0.72
NPV malaria test	0.99	0.85	0.98	0.85
NPV dengue test	0.91	0.98	0.91	0.91
NPV scrub typhus test	0.93	0.99	0.89	0.94
NPV typhoid test	0.98	1	0.92	0.99
NPV leptospirosis test	0.89	0.96	0.66	0.64

Abbreviations: NPV, negative predictive value; PPV, positive predictive value.

Using a simultaneous testing paradigm, the PPVs predicted by models ranged from 89% for dengue to 28% for typhoid in India, while in Cambodia the range was between 99% for malaria and 2% for typhoid. The highest negative predictive value (NPV) was for typhoid fever in both scenarios (98% and 100% for India and Cambodia, respectively) while applying all tests simultaneously. Sequential testing in the optimal algorithm largely improved the PPVs for many of the tests, the highest increase being observed for leptospirosis and typhoid tests in both the scenarios. Applying the tests sequentially decreased the NPVs of the tests, the highest decline predicted for the leptospirosis test.

### Optimal Algorithm and Correct Diagnosis

**Figure 2.**
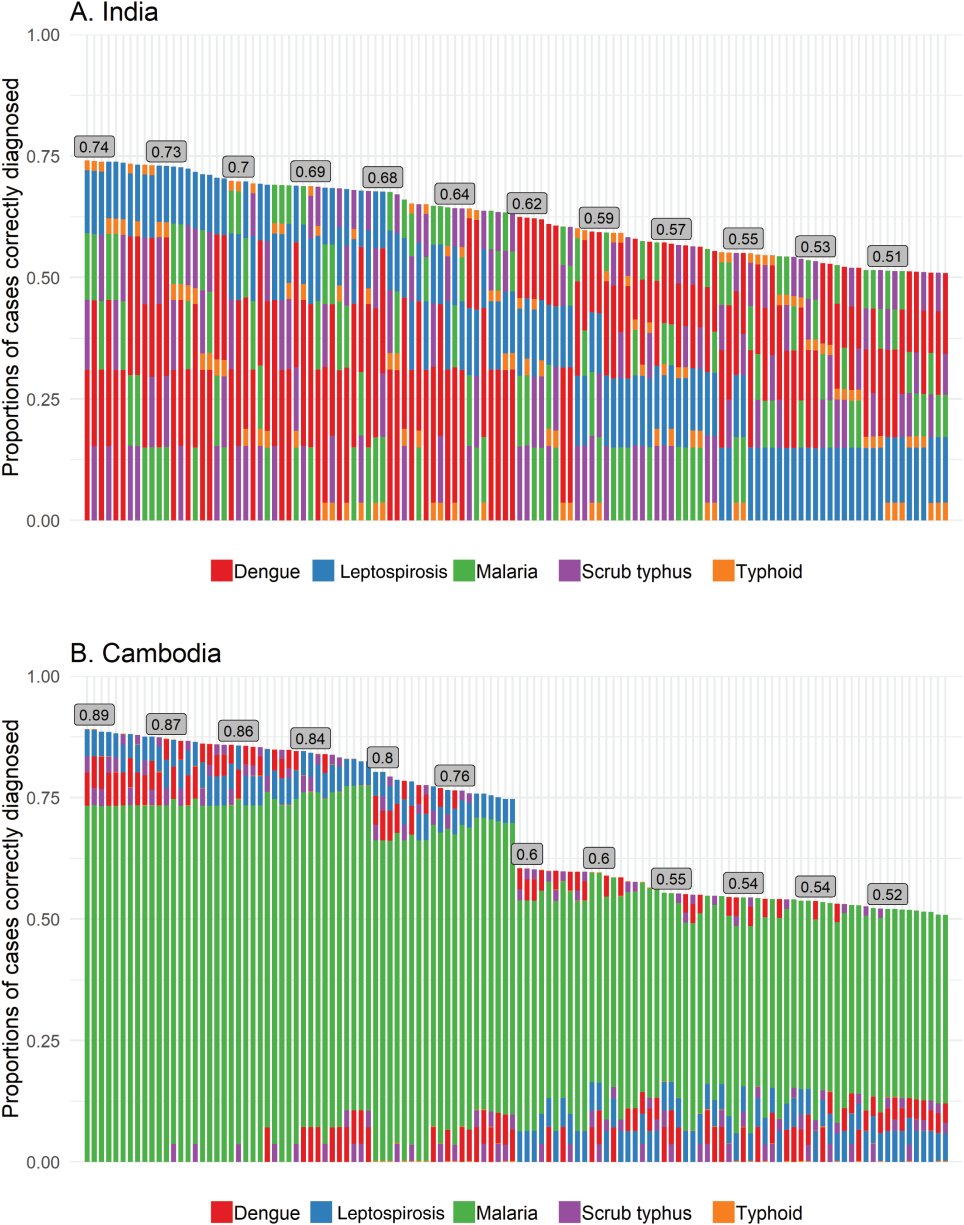
Algorithms with tests in different orders, for India (*A*) and Cambodia (*B*). Each column represents 1 of the 120 algorithms of 5 tests. The algorithms are arranged from left to right in decreasing correct diagnosis score. The stacked bars in each column represent the 5 tests that are performed in a sequential order, with the first test at the bottom of the column and the fifth test at the very top. The length of each bar represents the contribution of each test to total correct diagnosis score.

The 120 possible algorithms and the predicted correct diagnoses are shown in Figure 2.

The optimal algorithm for the application of these tests in India has the tests in the order of dengue, scrub typhus, malaria, leptospirosis, typhoid, with a correct diagnosis score of 0.74. The worst-performing algorithm has the tests in the order of typhoid, leptospirosis, malaria, scrub typhus, dengue, with an associated correct diagnosis score of 0.51. In Cambodia, the optimal algorithm has the tests in the order of malaria, dengue, scrub typhus, leptospirosis, typhoid, with a correct diagnosis score of 0.89. The worst performing algorithm has the tests in the order of typhoid, leptospirosis, scrub typhus, dengue, malaria, with a correct diagnosis score of 0.51.

Table 3 shows the average CD scores with 95% confidence intervals of algorithms based on the initial test in the algorithm, highlighting the importance of performing tests in the appropriate order for in a particular setting. The average CD score for algorithms starting with dengue was 0.68 in India compared with 0.65, 0.65, 0.60, and 0.53 for algorithms starting with malaria, scrub typhus, typhoid, and leptospirosis, respectively. Similarly, in the Cambodian setting, algorithms starting with malaria predicted a correct diagnosis score of 0.87 compared with 0.69, 0.69, 0.65, and 0.56 for algorithms starting with dengue, scrub typhus, typhoid, and leptospirosis, respectively.

**Table 3.** Average Correct Diagnosis Based on the Initial Test in the Algorithm

Tests at First Position in the Algorithm	India	Cambodia
	Mean CD Score (95% CI)	Mean CD Score (95% CI)
Malaria	0.65 (.62–.67)	0.87 (.86–.87)
Dengue	0.68 (.67–.70)	0.69 (.63–.74)
Scrub typhus	0.65 (.63–.68)	0.69 (.63–.75)
Typhoid	0.60 (.57–.62)	0.65 (.60–.70)
Leptospirosis	0.53 (.52–.54)	0.56 (.54–.57)

Abbreviations: CD, correct diagnosis; CI, confidence interval.

### Simplifying Algorithms

Algorithms with fewer tests were also explored, and the CD scores were predicted and compared with simultaneous testing (Table 4).

**Table 4.** Optimal and Worst Algorithms Using 1, 2, 3, and 4 of the 5 Tests With Corresponding Correct Diagnosis Score Using Currently Available Rapid Diagnostic Tests

No. of Tests	Optimal/Worst Algorithm	India			Cambodia		
		Order of Tests	CD Score With Sequential Testing	CD Score When Tests Are Applied Simultaneously	Order of Tests	CD Score With Sequential Testing	CD Score When Tests Are Applied Simultaneously
1	Optimal test	1. Dengue	0.31	0.31	1. Malaria	0.73	0.73
	Worst test	1. Typhoid	0.04	0.04	1. Typhoid	0.001	0.001
2	Optimal algorithm	1. Dengue 2. Scrub typhus	0.45	0.45	1. Malaria 2. Dengue	0.80	0.76
	Worst algorithm	1. Typhoid 2. Leptospirosis	0.17	0.16	1. Typhoid 2. Scrub typhus	0.03	0.03
3	Optimal algorithm	1.Dengue 2.Scrub typhus 3.Leptospirosis	0.59	0.42	1. Malaria 2. Dengue 3. Leptospirosis	0.86	0.55
	Worst algorithm	1.Typhoid 2.Leptospirosis 3.Malaria	0.26	0.24	1. Typhoid 2. Leptospirosis 3. Scrub typhus	0.08	0.08
4	Optimal algorithm	1.Dengue 2.Scrub typhus 3.Malaria 4.Leptospirosis	0.72	0.54	1. Malaria 2. Scrub typhus 3. Dengue 4. Leptospirosis	0.89	0.69
	Worst algorithm	1.Typhoid 2.Leptospirosis 3.Malaria 4.Scrub typhus	0.34	0.31	1. Typhoid 2. Leptospirosis 3. Scrub typhus 4. Dengue	0.12	0.11

Abbreviation: CD, correct diagnosis.

The model predicted the dengue test as the optimal single test to introduce first in the specified Indian setting, as it alone correctly diagnosed 31% of the patients. Similarly, malaria testing alone could correctly diagnose 73% of patients in the selected Cambodian setting. If 2 tests were to be applied, dengue followed by the scrub typhus test would predict the maximum correct diagnosis in 45% of patients in India, and malaria followed by the dengue test would predict a maximum correct diagnosis in 80% of patients in Cambodia, compared with 44% and 76% when these tests were applied simultaneously in India and Cambodia, respectively. The tests applied in the order of dengue, scrub typhus, and leptospirosis would predict the optimal diagnosis for any 3 tests in India, and correctly diagnose 59% of patients compared to 42% with tests applied simultaneously. Similarly, the tests applied in the order of malaria, dengue, and leptospirosis would diagnose 86% of patients correctly in Cambodia (compared to 55% with simultaneous testing). When 4 tests are available, the optimal algorithm in India would be dengue, scrub typhus, malaria, and leptospirosis and would correctly diagnose 72% of patients compared to 54% with simultaneous testing. Similarly, the algorithm with the order malaria, scrub typhus, dengue, and leptospirosis would correctly diagnose 89% of patients in Cambodia (compared to 69% with simultaneous testing).

## DISCUSSION

To enable better fever management in resource-limited settings, this work aims to identify suitable, geographically appropriate testing algorithms that can be implemented with the currently available diagnostic tests. The work intends to encourage health authorities and guide future research to institute diagnostic testing algorithms. Both simultaneous testing and sequential testing algorithms were explored to identify the most effective diagnostic approach to ensure that the clinical decisions made and resources allocated will be the most appropriate for improved patient care.

Simultaneous testing of either multiple single RDTs or in the form of a multiplex panel wrongly diagnosed a large number of patients in our model due to the suboptimal specificities of the tests. This type of testing, albeit common, results in ambiguity in interpretation of results and clinical decision making when a patient is positively diagnosed for >1 disease [20]. This may be appropriate for hospital settings where clinical expertise and diagnostic facilities are available for further differentiation of etiologies. However, at the primary healthcare level in a resource-poor setting, healthcare delivery largely depends on minimally trained healthcare workers who lack adequate clinical expertise to draw the responsible conclusions from the multiple positive results that may be seen on the testing panel [13]. Simultaneous testing for 5 common AUFIs, with the presently available RDTs in our model, correctly predicted the diagnosis of only roughly half of all patients in both settings, meaning that half of the patients are sent away without appropriate diagnosis and care, despite their extensive expenses on diagnostic testing. As a second approach, sequential testing in a rational order was found to increase the correct identification of the diseases and potentially decrease expenses. The sequential testing with multiple RDTs performed from the same blood sample circumvents the need for increase in labor and administrative complexities with multiple tests subjected to a patient. Also, reading the results in an appropriate algorithm rather than simultaneously of multiple RDT testing when performed together or with combined multiplex RDT testing correlates the optimal sequential testing and predicts better diagnosis.

Diagnostic tests in clinical practice are often arbitrarily applied in the order of presumed prevalence without considering test accuracy. The Widal test for typhoid is an example of a test that is widely available and often is the first test used in the diagnosis of AUFIs despite its limited accuracy [21]. However, the diagnostic outcome of an algorithm is very sensitive to the accuracies of component diagnostic tests. Correct identification of a component disease in an algorithm depends on the prevalence of the disease in the specific setting, and the diagnostic sensitivity and specificity of all prior tests in the algorithm. Our algorithmic approach to optimize the cumulative correct identification of the project AUFIs—taking into account the prevalence of all component diseases and their respective diagnostic test characteristics—predicted better diagnosis.

The large spatial heterogeneity in the etiologies of AUFIs [3], the effect of seasonality on their epidemiology [22], and nonuniform access to different RDTs limit the generalizability and global adoption of any algorithm. White et al compared the region-specific treatment algorithms with national empirical treatment protocols for AUFIs in the Lao People’s Democratic Republic and reported that a spatially explicit treatment algorithm based on local epidemiology significantly improves treatment outcomes [23]. The epidemiological data on AUFIs at the national and local levels are emerging across the world [2, 14, 24, 25]. The web application allows users to select their scenario using the best available epidemiological evidence and run an informal sensitivity analysis varying scenario parameters in choosing an optimally performing algorithms tailored to their setting. The order of tests in the top-performing algorithms may not have a huge effect on the correct identification of AUFIs, opening up an opportunity to deploy the same algorithm in different settings with similar causes of AUFIs. Ultimately, the decision to choose a particular algorithm among the top-performing ones to be made locally can be subjective taking into disease severity and availability to treatment as well as cost and availability of RDTs.

Undoubtedly, RDTs can provide valuable diagnostic support in low-resource settings where, due to the limited clinical expertise and healthcare infrastructure, patients are either not diagnosed at all or wrongly diagnosed based on empirical observation [26]. However, limited financial resources preclude the deployment of all available RDTs as a mere solution. Moreover, this study suggests that all tests may not necessarily offer substantial benefit in improving the overall diagnosis of the population, which is why a consideration of disease prevalence and test characteristics can inform cautious selection of the panel of tests required in a given setting for the most efficient use of scarce resources and the best possible treatment decisions.

Despite its utility, our model has several limitations. Most importantly, the model does not take into account coinfections and may therefore not completely represent real-life situations, particularly in high-malaria transmission areas [27]. The epidemiological data on coinfections of clinical significance is limited by the false positivity associated with tests deployed in the studies and asymptomatic carriers in the population not attributing to the disease. The web application allows users to explore the sensitivity of prevalence parameters tailored to the setting before application of an algorithm and is expected to sufficiently guide the utilization of tests. Likewise, the etiology of febrile illness differs largely across different age groups [2], which needs to be taken into account for more precise age-specific algorithms. The model also does not take into account disease severity and assumes that all the diseases under consideration have similar treatability and treatment outcomes. Missing a diagnosis for 1 disease etiology might lead to more severe clinical consequences as compared to others. It is important to not forget that diagnostic testing cannot happen in isolation and the final diagnosis by a clinician has to consider risk factors, clinical indicators, and measurable severity markers (eg. respiratory rate, lactate, procalcitonin) as additional precautions. Differentiating clinical signs and symptoms (eg, rose spots for typhoid, hemorrhagic rashes for dengue), although rare, should supersede diagnosis based on the order of testing in the algorithm when these symptoms are present and can be distinguished based on available clinical expertise and/or laboratory parameters, such as thrombocytopenia for dengue, for example. The prevalence estimates used in this study were derived from hospital-based studies, which may not represent a large rural population. The prevalence estimate used for malaria in Cambodia is unusually high [15], but is representative of a high malaria-endemic region. Also, the data on test accuracies were derived from the studies published from diverse settings and are not specific to our study settings. Despite our utmost efforts to use the best possible evidence, available estimates on disease prevalence and test accuracies may not accurately represent the true values in the study population and may therefore influence the results of this study.

It is widely recognized that available RDTs for AUFIs are insufficient and improved assays with superior diagnostic accuracy values are needed [9–11, 17]. The development of better tools demands substantial time and resources; thus, a new enhanced RDT may not become an immediately available solution. In the meantime, it is critical to take advantage of available tools and improve care using pragmatic yet evidence-based approaches. Using available tests based on appropriate algorithms is imperative and can substantially improve patient care decisions and ensure the most appropriate use of resources.

## Supplementary Data

Supplementary materials are available at *Clinical Infectious Diseases* online. Consisting of data provided by the authors to benefit the reader, the posted materials are not copyedited and are the sole responsibility of the authors, so questions or comments should be addressed to the corresponding author.

ciz665_suppl_Supplementary_Information

## Notes

***Author contributions.*** S. P., L. J. W., and S. D. conceived the study. S. P., L. J. W., and R. A. developed the model structure. S. P. and S. D. collected data for use in the model. S. P. performed the experiments. O. C. developed the web application for the model. S. P. wrote the first draft. L. J. W., R. A., O. C., K. P., and S. D. assisted in interpretation of results and revision of the manuscript. All authors read and approved the final version of the manuscript.

***Acknowledgments.*** The authors acknowledge the invaluable advice and support from Professor Brian Angus and Dr Proochista Ariana at the Centre for Tropical Medicine and Global Health, Nuffield Department of Medicine, University of Oxford, United Kingdom. The authors are grateful to Dr Stefano Ongarello, Dr Seda Yerlikaya, and Zachary Katz at the Foundation for Innovative New Diagnostics (FIND) for their input. The authors also thank Beatrice Gordis for her editorial support.

***Financial support.*** FIND’s work on malaria and fever is funded by UK aid from the British people and received backing from the Fondation Botnar. This work was supported by the Bill & Melinda Gates Foundation (grant number OPP1110500) and was part of the Wellcome Trust–Mahidol Oxford Tropical Medicine Research Unit, funded by the Wellcome Trust of Great Britain (grant number 101148/Z/13/Z). The initial analysis formed the basis of the dissertation project undertaken by S. P., funded by the MSc in International Health and Tropical Medicine programme at the Nuffield Department of Medicine, University of Oxford.

***Potential conflicts of interest.*** The authors report no potential conflicts of interest. All authors have submitted the ICMJE Form for Disclosure of Potential Conflicts of Interest. Conflicts that the editors consider relevant to the content of the manuscript have been disclosed.
