# Supplementary material for: Algorithm in the Diagnosis of Febrile Illness Using Pathogen-specific Rapid Diagnostic Tests
Source: Clin Infect Dis. 2019 Jul 17;70(11):2262–9. doi: 10.1093/cid/ciz665 (PMC7245147; doi:10.1093/cid/ciz665)
Supplement: ciz665_suppl_Supplementary_Information [file ciz665_suppl_supplementary_information.pdf]

Algorithm in the diagnosis of Febrile Illness using pathogen-specific Rapid Diagnostic Tests

Supplementary materials:

Authors: Sunil Pokharel, Lisa J White, Ricardo Aguas, Olivier Celhay, Karell G Pellé, Sabine Dittrich

Supplementary contents:

1. **Equations for simultaneous testing.......... 2**
2. **Equations for sequential testing.......... 3**
3. **Correct diagnosis.......... 4**
4. **R script for simultaneous testing.......... 5**
5. **R script for sequential testing.......... 10**

Equations: Simultaneous testing

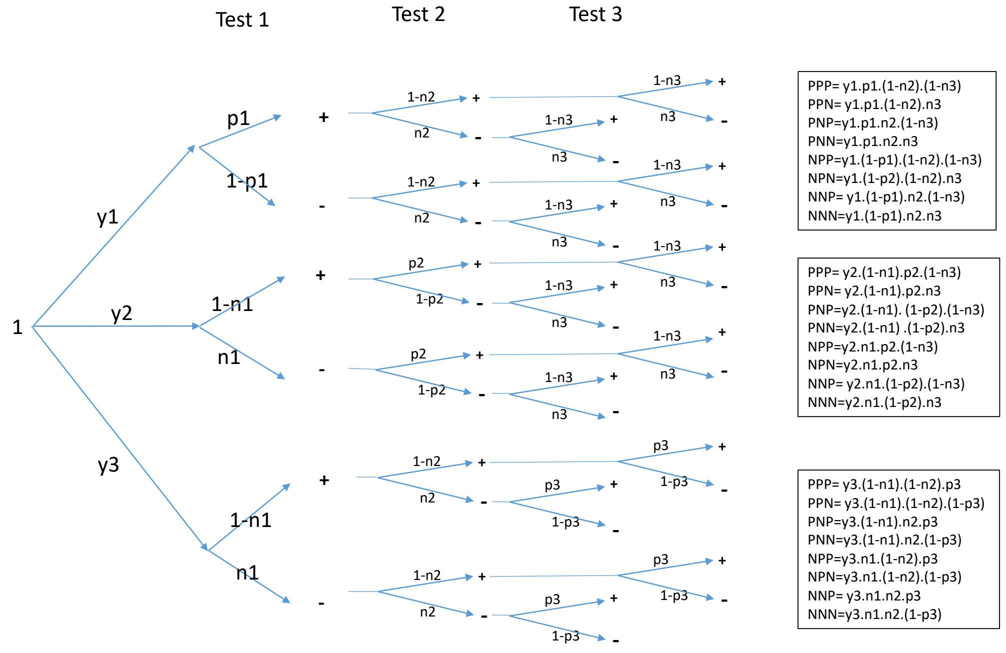

Decision tree for simultaneous testing
(Here y1, y2, y3 are relative prevalence of disease 1, disease 2 and disease 3 respectively,
Test 1, Test 2, Test 3 are tests for disease 1, disease 2 and disease 2 respectively,
p1, p2, p3 are respective sensitivities and n1, n2 and n3 are respective specificities for Test 1, Test 2 and Test 3)

<u>Simplified equations:</u>
For k diseases with prevalence given by y1, y2, y3… yk, sensitivity of test for respective disease given by p1, p2, p3….pk and corresponding specificities given by n1, n2, n3…..nk, different diagnostic outputs are calculated as follows:

<u>Total True Positives</u>
TP = y1.p1 + y2.p2 + y3.p3 +…….. yk.pk

$$TP = \sum_{i=1}^{k} y_i . p_i$$

<u>Total False Positives</u>
FP = (1-y1).(1-n1) + (1-y2).(1-n2) + (1-y3).(1-n3) + ……… (1-yk).(1-nk)
`

$$FP = \sum_{i=1}^{k} (1 - y_i).(1 - n_i)$$

<u>Total True Negatives</u>
TN = (1-y1).n1 + (1-y2).n2 + (1-y3).n3 + …. (1-yk).nk

$$TN = \sum_{i=1}^{k} (1 - y_i).n_i$$

Total False Negatives

FN = y1.(1-p1) + y2.(1-p2) + y3.(1-p3) + …. yk.(1-pk)

$$FN = \sum_{i=1}^{k} y_i.(1 - p_i)$$

Equations: Sequential testing

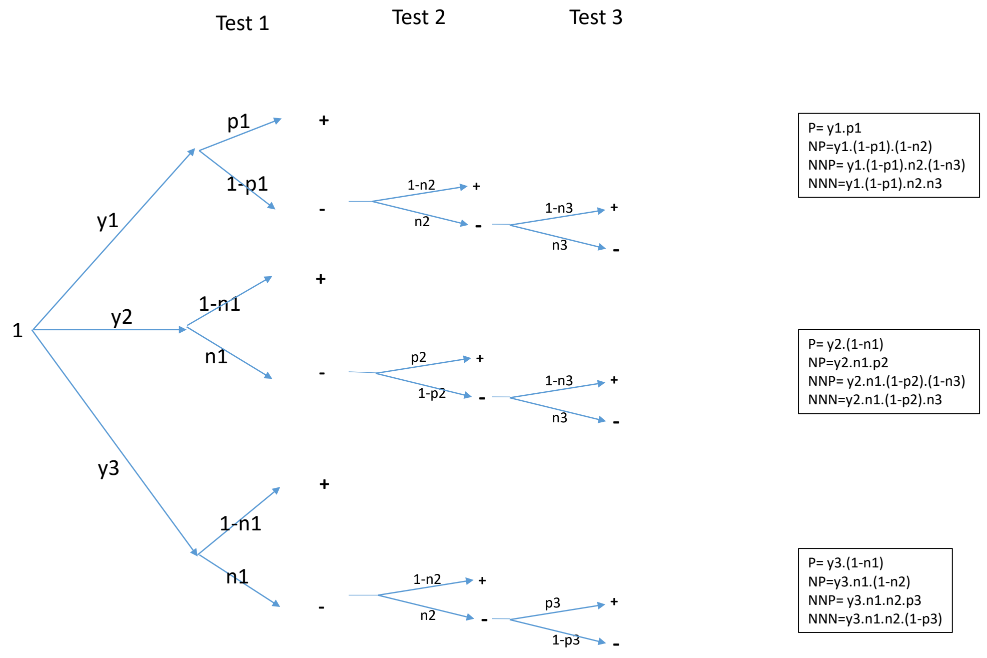

Figure 1: Decision tree for sequential testing
(Here y1, y2, y3 are relative prevalence of disease 1, disease 2 and disease 3 respectively,
Test 1, Test 2, Test 3 are tests for disease 1, disease 2 and disease 2 respectively,
p1, p2, p3 are respective sensitivities and n1, n2 and n3 are respective specificities for Test 1, Test 2 and Test 3)

Simplified equations:

Total True Positives (Correct diagnosis score)

TP = y1.p1 + y2.n1.p2 + y3.n1.n2.p3 ……

$$TP = \sum_{i=1}^{k} \frac{y_i p_i \prod_{j=1}^{i} n_j}{n_i}$$

Total False Positives

FP = (y2 + y3).(1-n1) + y1.(1-p1).(1-n2) + y3.n1.(1-n2) + y1.(1-p1).n2.(1-n3) + y2.n1.(1-p2).(1-n3) +....

$$FP = \sum_{i=2}^{k}[\ \prod_{m=1}^{i-1} n_m.(1-n_i)\sum_{j=1}^{i-1}\frac{y_j(1-p_j)}{n_j}] +\ \sum_{i=1}^{k}[\sum_{j=i+1}^{k}\frac{y_j(1-n_i)\prod_{m=1}^{i} n_m}{n_i}\ ]$$

<u>Total True Negatives</u>
TN = (y2+y3).n1 + y1.(1-p1).n2 + y3.n1.n2. + y1.(1-p1).n2.n3 + y2.n1.(1-p2).n3 + ...

$$TN = \sum_{i=2}^{k}[\ \prod_{m=1}^{i} n_m.\sum_{j=1}^{i-1}\frac{y_j(1-p_j)}{n_j}] +\ \sum_{i=1}^{k}[\sum_{j=i+1}^{k} y_j \prod_{m=1}^{i} n_m\ ]$$

<u>Total False Negatives</u>
FN = y1.(1-p1) + y2.n1.(1-p2) + y3.n1.n2.(1-p3)+ ......

$$FN = \sum_{i=1}^{k}\frac{y_i(1-p_i)\prod_{j=1}^{i} n_j}{n_i}$$

Correct diagnosis:

Correct diagnosis of each disease while tested in algorithm:

$$CDi = \frac{y_i p_i \prod_{j=1}^{i} n_j}{n_i}$$

Correct diagnosis of each disease while tested simultaneously

$$CDi = \frac{y_i p_i \prod_{j=1}^{k} n_j}{n_j}$$

Total correct diagnosis while tested in algorithm:

$$CD = \sum_{i=1}^{k}\frac{y_i p_i \prod_{j=1}^{i} n_j}{n_i}$$

Total correct diagnosis of while tested simultaneously

$$CD = \sum_{i=1}^{k}\frac{y_i p_i \prod_{j=1}^{k} n_j}{n_j}$$

The correct diagnosis of each disease in simultaneous testing is thus the function of relative prevalence of the disease, sensitivity of test for the disease and specificities of all other tests. Similarly, the correct diagnosis of each disease in sequential testing is the function of prevalence of the disease, sensitivity of test for the disease and specificity of all prior tests in the algorithm. This implies that specificity of a component test affects the correct diagnosis of all other diseases in simultaneous testing but influences only the proceeding diagnoses in the sequential testing algorithms.

R script for simultaneous testing

```
#######################################################
# Program for simultaneous testing of febrile illnesses#
#######################################################
# the code uses DescTools and matrixStats packages and for loop function

#call packages
library(DescTools)
library(matrixStats)

y<-100                    #determine the number of trials

#Initialize output
OUTpropCD<-matrix(NA, y, 5) #proportion of correct diagnosis for each diagnosis
OUTsumCD<-c()              #total correct diagnosis
OUTPPV<-matrix(NA, y, 5)   #positive predictive value for each disease diagnosis
OUTNPV<-matrix(NA, y, 5)   #negative predictive value for each disease diagnosis

for (k in 1:y)           #repeats the run for trial
{
#model inputs
pv<- c(3, 7, 4, 1, 4)             #prevalance: Malaria, Dengue, Scrub, Typhoid, Lepto in India
#pv<- c(31.8, 3.5, 2.1, 0.1, 3.7)   # prevalence: : Malaria, Dengue, Scrub, Typhoid, Lepto in Cambodia
pvx<-0                            #Other prevalance
sn<- c(0.95, 0.842, 0.728, 0.69, 0.71)   #Sensitivities of malaria, dengue, scrub, typhoid and lepto test
sp<- c(0.952, 0.94, 0.968, 0.9, 0.646)   #Specifities of malaria, dengue, scrub, typhoid and lepto test
rp<- pv/(sum(pv)+pvx)            #relative prevalances

#cumulative relative prevalance of five diseases
crp1<- rp[1]
crp2<- crp1+rp[2]
crp3<- crp2+rp[3]
crp4<- crp3+rp[4]
crp5<- crp4+rp[5]
crp<-c(crp1, crp2, crp3, crp4, crp5)

x<-10000                  #determine the number of simulations
rand<-runif(x,0,1)        #generate random number for each simulation

#Assign diagnosis based on relative prevalance
ds<-c()
for(i in 1:x)
{
  if(rand[i]<=crp[1])
  {ds[i]<-1
  }
  else if(rand[i]<=crp[2])
  {ds[i]<-2
  }
  else if(rand[i]<=crp[3])
  {ds[i]<-3
  }
  else if(rand[i]<=crp[4])
  {ds[i]<-4
  }
  else if(rand[i]<=crp[5])
```

```
  {ds[i]<-5
  }
  else{ds[i]<-0}
}
dis<- c(sum(ds==1),sum(ds==2), sum(ds==3), sum(ds==4), sum(ds==5))    #total pretest diagnosis of five diseases

#TP for first test
TP1<-c()
for(i in 1:x)
{
  if(ds[i]==1 & runif(1)<=sn[1])
  {TP1[i]<-1
  }
  else {TP1[i]<-0}
}

#FP for first test
FP1<-c()
for(i in 1:x)
{
  if(ds[i]!=1 & runif(1)<=(1-sp[1]))
  {FP1[i]<-1
  }
  else{FP1[i]<-0
  }
}

#TP for second test
TP2<-c()
for(i in 1:x)
{
  if(ds[i]==2 & runif(1)<=sn[2])
  {TP2[i]<-1
  }
  else {TP2[i]<-0}
}

#FP for second test
FP2<-c()
for(i in 1:x)
{
  if(ds[i]!=2 & runif(1)<=(1-sp[2]))
  {FP2[i]<-1
  }
  else{FP2[i]<-0
  }
}

#TP for third test
TP3<-c()
for(i in 1:x)
{
  if(ds[i]==3 & runif(1)<=sn[3])
  {TP3[i]<-1
  }
  else {TP3[i]<-0}
```

```
}

#FP for third test
FP3<-c()
for(i in 1:x)
{
  if(ds[i]!=3 & runif(1)<=(1-sp[3]))
  {FP3[i]<-1
  }
  else{FP3[i]<-0
  }
}

#TP for forth test
TP4<-c()
for(i in 1:x)
{
  if(ds[i]==4 & runif(1)<=sn[4])
  {TP4[i]<-1
  }
  else {TP4[i]<-0}
}

#FP for forth test
FP4<-c()
for(i in 1:x)
{
  if(ds[i]!=4 & runif(1)<=(1-sp[4]))
  {FP4[i]<-1
  }
  else{FP4[i]<-0
  }
}

#TP for fifth test
TP5<-c()
for(i in 1:x)
{
  if(ds[i]==5 & runif(1)<=sn[5])
  {TP5[i]<-1
  }
  else {TP5[i]<-0}
}

#FP for fifth test
FP5<-c()
for(i in 1:x)
{
  if(ds[i]!=5 & runif(1)<=(1-sp[5]))
  {FP5[i]<-1
  }
  else{FP5[i]<-0
  }
}
sumTP<- c(sum(TP1), sum(TP2), sum(TP3), sum(TP4), sum(TP5))          #Total true positives for each of five diseases
```

```
sumFP<- c(sum(FP1), sum(FP2), sum(FP3), sum(FP4), sum(FP5))       #total false positives for each of five diseases
sumToP<- sumTP+sumFP            #total positives for five diseases
sumTN<-x-dis-sumFP              #true negative for five diseases
sumFN<-dis-sumTP                #true negative for five diseases

#calcuation of total false postives
FP<-c()
for(i in 1:x)
{
  if(FP1[i]==1 | FP2[i]==1 | FP3[i]==1 | FP4[i]==1 | FP5[i]== 1)
  {FP[i]<-1
  }
  else{FP[i]<-0
  }
}
sum(FP)

#calculation of correct diagnosis
CD<-c()
for (i in 1:x)
{if (TP1[i]==1 & FP[i]==0)
{CD[i]<-1
}
  else if (TP2[i]==1 & FP[i]==0)
  {CD[i]<-2
  }
  else if (TP3[i]==1 & FP[i]==0)
  {CD[i]<-3
  }
  else if (TP4[i]==1 & FP[i]==0)
  {CD[i]<-4
  }
  else if (TP5[i]==1 & FP[i]==0)
  {CD[i]<-5
  }
  else {CD[i]<-0
  }
}

#Outcome calculations
sumCD<-c(sum(CD==1), sum(CD==2), sum(CD==3), sum(CD==4), sum(CD==5))       #number of each cases correctly diagnosed
ssumCD<-sum(sumCD)/x                          #proportion of all cases correctly diagnoed
propCD<-sumCD/dis                           ##proportion of each cases correctly diagnosed
PPV<- sumTP/sumToP                          #PPV for each test
NPV<- sumTN/(sumTN+sumFN)                          #NPV for each test
#Defining model output
OUTpropCD[k,]<-propCD
OUTsumCD[k]<-ssumCD
OUTPPV[k,]<-PPV
OUTNPV[k,]<-NPV
}
#dataframe of outputs
d1<- data.frame(OUTsumCD, OUTpropCD, OUTPPV, OUTNPV)
##calculation of mean outputs from all trials
#Total correct diagnosis, mean and CI
```

```
meansumCD<-mean(OUTsumCD)
#mean proportion of correct diagnosis
meanpropCD<-colMeans2(OUTpropCD, na.rm=FALSE)
#mean PPV for individual tests
meanPPV<- colMeans2(OUTPPV, na.rm=FALSE)
#mean PPV for individual tests
meanNPV<- colMeans2(OUTNPV, na.rm=FALSE)
#Outputs
meansumCD    #proportion of ovearall correct diagnosis
meanpropCD   #proportion of correct diagnosis by case
meanPPV      #positive predictive value by case
meanNPV       #negative predictive value by case
```

R script for sequential testing

```
#######################################################
# Program do develop sequential testing algorithm for febrile illness #
#######################################################
# the code uses gtools, data.table and ggpplot2, matrixStats, dplyr and blindrcpp packages, permutataion and for loop functions.

#call packages
library(gtools)
library(data.table)
library(ggplot2)
library(matrixStats)
library(dplyr)
library(bindrcpp)

# create all possible combinations of tests
n<- 5 #number of tests
r<- 5 #number of disease
perm<-permutations(n, r, v = 1:n)  #possible combinations

lp<- length(perm[,1])  #length of permutation vector

# initialise output
OUTpropCD<-matrix(NA, lp, r) #proportion of correct diagnosis for each diagnosis
OUTsumCD<-c()              #total correct diagnosis
OUTPPV<-matrix(NA, lp, r)    #positive predictive value for each test
OUTNPV<-matrix(NA, lp, r)    #negative predictive value for each test
OUTCD<-matrix(NA, lp, r)     #each test contribution to total correct diagnosis
OUTCCD<-matrix(NA, lp, r)    #cumulative correct diagnosis after every test in algorithm

#Model inputs
pv<- c(3, 7, 4, 1, 4)#prevalance of malaria, dengue, scrub, typhoid and lepto (India)
#pv<- c(31.8, 3.5, 2.1, 0.1, 3.7) #prevalance of malaria, dengue, scrub, typhoid and lepto (India)
pvx<-0             #other prevalance
sn<- c(0.95, 0.842, 0.728, 0.69, 0.71) #corresponding sensitivity
sp<- c(0.952, 0.94, 0.968, 0.9, 0.646) #corresponding specificity

for (j in 1:lp)   #repeats the run for each of 120 possible algorithm
{
  pvj<-pv[perm[j,]]
  snj<-sn[perm[j,]]
  spj<-sp[perm[j,]]

  #Define the number of trials for each algorithm
  y<-100

  #Initialize output
  OpropCD<-matrix(NA, y, 5) #proportion of correct diagnosis for each disease
  OsumCD<-c()              #total correct diagnosis
  OPPV<-matrix(NA, y, 5)    #positive predictive value for each disease disease
  OsumPPV<-c()              #overall negative predictive value for the whole algorithm
  ONPV<-matrix(NA, y, 5)    #negative predictive value for each disease
  OsumNPV<-c()              #overall positive predictive value for the whole algorithm
  OCCD<-matrix(NA, y, 5)    #cumulative correct diagnosis
  OCD<-matrix(NA, y, 5)     #correct diagnosis for each disease
```

```
for (k in 1:y)            #repeat the simulation for each trial
{
x<-10000  #number of simulations
rand<-runif(x,0,1)  #10000 random numbers between 0 and 1

  ds <- rep(0, x)
  TP1 <- rep(0, x)
  FP1 <- rep(0, x)
  TP2 <- rep(0, x)
  FP2 <- rep(0, x)
  TP3 <- rep(0, x)
  FP3 <- rep(0, x)
  TP4 <- rep(0, x)
  FP4 <- rep(0, x)
  TP5 <- rep(0, x)
  FP5 <- rep(0, x)
  TN1 <- rep(0, x)
  FN1 <- rep(0, x)
  TN2 <- rep(0, x)
  FN2 <- rep(0, x)
  TN3 <- rep(0, x)
  FN3 <- rep(0, x)
  TN4 <- rep(0, x)
  FN4 <- rep(0, x)
  TN5 <- rep(0, x)
  FN5 <- rep(0, x)
  CD1 <- rep(0, x)
  CD2 <- rep(0, x)
  CD3 <- rep(0, x)
  CD4 <- rep(0, x)
  CD5 <- rep(0, x)

  pvj <- pv[perm[j, ]]
  snj <- sn[perm[j, ]]
  spj <- sp[perm[j, ]]

  rp <- pvj/(sum(pvj)+pvx)    #relative prevalance
  crp <- cumsum(rp) #cumulative relative prevalance

  rand<-runif(x, 0, 1)  #x random numbers between 0 and 1; each random generates a patient

  rand_unif <- matrix(runif(10*x, 0, 1), x, 10)

  for(i in 1:x)
  {
    #Assign diagnosis based on pretest probability
    v <- which(rand[i] <= crp)
    if(any(v)) ds[i] <- min(v)
  }

  TP1[ds == 1 & rand_unif[, 1] <= snj[1]] <- 1
  FP1[ds != 1 & rand_unif[, 2] <= (1 - spj[1])] <- 1
  TP2[ds == 2 & TP1 == 0 & FP1 == 0 & rand_unif[, 3]<=snj[2]] <- 1
  FP2[ds != 2 & TP1 == 0 & FP1 == 0 & rand_unif[, 4]<=(1-spj[2])] <- 1
  TP3[ds == 3 & TP1 == 0 & FP1 == 0 & TP2 == 0 & FP2 == 0 & rand_unif[, 5]<=snj[3]] <- 1
  FP3[ds != 3 & TP1 == 0 & FP1 == 0 & TP2 == 0 & FP2 == 0 & rand_unif[, 6]<=(1-spj[3])] <- 1
```

```
   TP4[ds == 4 & TP1 == 0 & FP1 == 0 & TP2 == 0 & FP2 == 0 & TP3 == 0 & FP3 == 0 & rand_unif[, 7]<=snj[4]] <- 1
   FP4[ds != 4 & TP1 == 0 & FP1 == 0 & TP2 == 0 & FP2 == 0 & TP3 == 0 & FP3 == 0 & rand_unif[, 8]<=(1-spj[4])] <- 1
   TP5[ds == 5 & TP1 == 0 & FP1 == 0 & TP2 == 0 & FP2 == 0 & TP3 == 0 & FP3 == 0 & TP4 == 0 & FP4 == 0 & rand_unif[, 9]<=snj[5]] <- 1
   FP5[ds != 5 & TP1 == 0 & FP1 == 0 & TP2 == 0 & FP2 == 0 & TP3 == 0 & FP3 == 0 & TP4 == 0 & FP4 == 0 & rand_unif[, 10]<=(1-spj[5])] <- 1

   TN1[ds != 1 & FP1 == 0] <- 1
   FN1[ds == 1 & TP1 == 0] <- 1
   TN2[ds != 2 & TP1 == 0 & FP1 == 0 & FP2 == 0] <- 1
   FN2[ds == 2 & TP1 == 0 & FP1 == 0 & TP2 == 0] <- 1
   TN3[ds != 3 & TP1 == 0 & FP1 == 0 & TP2 == 0 & FP2 == 0 & FP3 == 0] <- 1
   FN3[ds == 3 & TP1 == 0 & FP1 == 0 & TP2 == 0 & FP2 == 0 & TP3 == 0] <- 1
   TN4[ds != 4 & TP1 == 0 & FP1 == 0 & TP2 == 0 & FP2 == 0 & TP3 == 0 & FP3 == 0 & FP4 == 0] <- 1
   FN4[ds == 4 & TP1 == 0 & FP1 == 0 & TP2 == 0 & FP2 == 0 & TP3 == 0 & FP3 == 0 & TP4 == 0] <- 1
   TN5[ds != 5 & TP1 == 0 & FP1 == 0 & TP2 == 0 & FP2 == 0 & TP3 == 0 & FP3 == 0 & TP4 == 0 & FP4 == 0 & FP5 == 0] <- 1
   FN5[ds == 5 & TP1 == 0 & FP1 == 0 & TP2 == 0 & FP2 == 0 & TP3 == 0 & FP3 == 0 & TP4 == 0 & FP4 == 0 & TP5 == 0] <- 1

   CD1[TP1==1]<-1
   CD2[TP2==1]<-1
   CD3[TP3==1]<-1
   CD4[TP4==1]<-1
   CD5[TP5==1]<-1

  dis<- c(sum(ds==1),sum(ds==2), sum(ds==3), sum(ds==4), sum(ds==5) )  # QUESTION: Should you not record the case ds == 0? (Ans: not required at this time)

  sumTP<- c(sum(TP1), sum(TP2), sum(TP3), sum(TP4), sum(TP5))     #total true positives for each test

  sumFP<- c(sum(FP1), sum(FP2), sum(FP3), sum(FP4), sum(FP5))     #total false postives for each test

  sumToP<- sumTP+sumFP                                  #total positives for each test

  sumTN<- c(sum(TN1), sum(TN2), sum(TN3), sum(TN4), sum(TN5))     #total true negatives for each test

  sumFN<- c(sum(FN1), sum(FN2), sum(FN3), sum(FN4), sum(FN5))      #total false negatives


  #Outcome calculations for each run

  sumCD<-c(sum(CD1), sum(CD2), sum(CD3), sum(CD4), sum(CD5))     #number of correct diagnosis for each test

  #Cumulative correct diagnosis (proportion)
  CCD<- c(sumCD[1]/x, (sumCD[1]+sumCD[2])/x, (sumCD[1]+sumCD[2]+sumCD[3])/x,
        (sumCD[1]+sumCD[2]+sumCD[3]+sumCD[4])/x,
(sumCD[1]+sumCD[2]+sumCD[3]+sumCD[4]+sumCD[5])/x)

  #proportion of each conditions correctly diagnosed (denominator= each disease)
  propCD<-sumCD/dis

  #contribution of each test to correct diagnosis (denominator= total population)
  sCD<- sumCD/x
```

```
  #proportions of all cases correctly diagnosed
  ssumCD<- sum(sumCD)/x

  #predictive values for every test in each algorithm
  PPV<- sumTP/sumToP
  NPV<- sumTN/(sumTN+sumFN)

  #Define outputs each simulation
  OpropCD[k,]<-propCD              #proportion of each cases correctly diagnosed
  OsumCD[k]<-ssumCD                #proportion of all cases correctly diagnosed
  OPPV[k,]<-PPV                   #PPV of each test
  ONPV[k,]<-NPV                   #NPV of each test
  OCCD[k,]<-CCD                   #cumulative correct diagnosis after each test
  OCD[k,]<-sCD                   #each test contribution to correct diagnosis
  }

  #calulate mean outputs from trials
  meansumCD<-mean(OsumCD)             #mean total correct diagnosis score

  meanpropCD<-colMeans2(OpropCD, na.rm=FALSE)        #mean proportion of correct diagnosis

  meanPPV<- colMeans2(OPPV, na.rm=FALSE)     #mean PPV for individual tests

  meanNPV<- colMeans2(ONPV, na.rm=FALSE)     #mean NPV for individual tests

  meanCCD<- colMeans2(OCCD, na.rm=FALSE)    #mean cumulative correct diagnosis

  meanCD<- colMeans2(OCD, na.rm=FALSE)       #mean of each test contribution to correct diagnosis

  #Define Output for each combination of test
  OUTpropCD[j,]<-meanpropCD
  OUTsumCD[j]<-meansumCD
  OUTPPV[j,]<-meanPPV
  OUTNPV[j,]<-meanNPV
  OUTCCD[j,]<-meanCCD
  OUTCD[j,]<-meanCD
}

#data frame of required data
df<-data.frame(perm, OUTsumCD, OUTCD, OUTCCD, OUTpropCD, OUTPPV, OUTNPV)
#df
df1<-df[order(df$OUTsumCD, decreasing = TRUE),] #orders dataframe in descending order of total correct diagnosis

#rename column headings
setnames(df1, old = c('X1','X2','X3','X4','X5','OUTsumCD','X1.2','X2.2','X3.2','X4.2',
                    'X5.2'), new = c('test1','test2', 'test3','test4',
                    'test5', 'correctly diagnosed','Cum dx1','Cum dx2','Cum dx3','Cum dx4',
                    'Cum dx5'))
df1$test1[df1$test1==1]<-"Malaria"
df1$test1[df1$test1==2]<-"Dengue"
df1$test1[df1$test1==3]<-"Scrub typhus"
df1$test1[df1$test1==4]<-"Typhoid"
df1$test1[df1$test1==5]<-"Leptospira"
df1$test2[df1$test2==1]<-"Malaria"
```

```
df1$test2[df1$test2==2]<-"Dengue"
df1$test2[df1$test2==3]<-"Scrub typhus"
df1$test2[df1$test2==4]<-"Typhoid"
df1$test2[df1$test2==5]<-"Leptospira"
df1$test3[df1$test3==1]<-"Malaria"
df1$test3[df1$test3==2]<-"Dengue"
df1$test3[df1$test3==3]<-"Scrub typhus"
df1$test3[df1$test3==4]<-"Typhoid"
df1$test3[df1$test3==5]<-"Leptospira"
df1$test4[df1$test4==1]<-"Malaria"
df1$test4[df1$test4==2]<-"Dengue"
df1$test4[df1$test4==3]<-"Scrub typhus"
df1$test4[df1$test4==4]<-"Typhoid"
df1$test4[df1$test4==5]<-"Leptospira"
df1$test5[df1$test5==1]<-"Malaria"
df1$test5[df1$test5==2]<-"Dengue"
df1$test5[df1$test5==3]<-"Scrub typhus"
df1$test5[df1$test5==4]<-"Typhoid"
df1$test5[df1$test5==5]<-"Leptospira"

#algorithm for best correct diagnosis
a1<-which(OUTsumCD==max(OUTsumCD))

#Plot all algorithms with contribution of each test to correct diagnosis
d1 <- data.frame(Algorithms=factor(paste0("", 1:120),
                                  levels =rev(paste0("", 1:120))), data.frame(df1$test1,
                                          df1$test2, df1$test3, df1$test4, df1$test5))
d1m <- melt(d1, id.var = 'Algorithms')
d2<-data.frame(Algorithm=factor(paste0("", 1:120), levels =(paste0("", 1:120))), df1$X1.1,
              df1$X2.1, df1$X3.1, df1$X4.1, df1$X5.1)
d2M<-melt(d2, id.vars="Algorithm")
d2m<- data.table(d2M$Algorithm, d1m$variable, d1m$value, d2M$value)
d2m$CD<-df1$`correctly diagnosed`
d2m$rank_test <- substr(d2m$V2, start = 9, stop = 10)
d2m$rank_test <- factor(d2m$rank_test)
d2m$V3 <- as.character(d2m$V3)
p2 <- ggplot(d2m, aes(x = V1, y = V4, order = rev(rank_test), fill = V3)) +
  geom_bar(position = "stack", stat = "identity", width=0.7) +
  geom_label(data = d2m %>%
             filter(row_number() %% 10 == 2),
            aes(y = CD, label = round(CD, 2)), fill = 'grey', nudge_y = 0.03) +
  scale_fill_brewer(palette = "Set1") +
  scale_y_continuous(limits = c(0, 1), expand = c(0, 0)) +
  scale_x_discrete(expand = c(0.02, 0)) +
  labs(title = "A. India", x = NULL,
       y = "Proportions of cases correctly diagnosed") +
  theme_minimal(base_size = 16) +
  theme(axis.text.x = element_blank(), axis.ticks.x = element_blank(),
        legend.position = "bottom", legend.text = element_text(size = 14,
          margin = margin(r = 0.2, unit = 'in')), legend.title = element_blank())
p2
```
